# Tract-Specific White Matter Correlates of Age-Related Reward Devaluation Deficits in Macaque Monkeys

**DOI:** 10.17756/jnpn.2018-023

**Published:** 2018-07-19

**Authors:** Daniel T. Gray, Lavanya Umapathy, Sara N. Burke, Theodore P. Trouard, Carol A. Barnes

**Affiliations:** 1 Division of Neural System, Memory & Aging, University of Arizona, Tucson, AZ, USA; 2 Evelyn F McKnight Brain Institute, University of Arizona, Tucson, AZ, USA; 3 Electrical and Computer Engineering, University of Arizona, Tucson, AZ, USA; 4 Evelyn F McKnight Brain Institute, University of Florida, Gainesville, FL, USA; 5 Department of Biomedical Engineering, University of Arizona, Tucson, AZ, USA; 6 Departments of Psychology, Neurology and Neuroscience, University of Arizona, Tucson, AZ, USA

**Keywords:** Orbitofrontal cortex, Basolateral amygdala, Reward devaluation, Reversal learning, MRI, DTI, Probabilistic tractography

## Abstract

**Aim:**

Cognitive aging is known to alter reward-guided behaviors that require interactions between the orbitofrontal cortex (OFC) and amygdala. In macaques, OFC, but not amygdala volumes decline with age and correlate with performance on a reward devaluation (RD) task. The present study used diffusion magnetic resonance imaging (dMRI) methods to investigate whether the condition of the white matter associated with amygdala-OFC connectivity changes with age and relates to reward devaluation.

**Methods:**

Diffusion-, T1- and T2-weighted MRIs were acquired from adult and aged bonnet macaques. Using probabilistic tractography, fractional anisotropy (FA) estimates from two separate white matter tracts associated with amygdala-OFC connectivity, the uncinate fasciculus (UF) and amygdalofugal (AF) pathways, were obtained. Performance measures on RD and reversal learning (RL) tasks were also acquired and related to FA indices from each anatomical tract.

**Results:**

Aged monkeys were impaired on both the RD and RL tasks and had lower FA indices in the AF pathway. Higher FA indices from the right hemisphere UF pathway correlated with better performance on an object-based RD task, whereas higher FA indices from the right hemisphere AF were associated with better performance on an object-free version of the task. FA measures from neither tract correlated with RL performance.

**Conclusions:**

These results suggest that the condition of the white matter connecting the amygdala and OFC may impact reward devaluation behaviors. Furthermore, the observation that FA indices from the UF and AF differentially relate to reward devaluation suggests that the amygdala-OFC interactions that occur via these separate tracts are partially independent.

## Introduction

The ability to revalue reinforced stimuli according to changing biological or psychological needs is a critical component of adaptive, reward-driven behaviors since it affords animals the ability to select advantageous action plans when presented with competing cues. Nonhuman primate models of cognitive aging show devaluation deficits such that younger individuals tend to behave in a manner that optimizes reward value to a greater extent than do older individuals [1]. The most commonly used behavioral assay to test this cognitive function in monkeys is the reinforcer devaluation task [2, 3], which tests an animal’s ability to avoid stimuli associated with food rewards that had recently been consumed to satiety (i.e., devalued) when presented alongside stimuli associated with relatively non-devalued reinforcement. Lesion studies have implicated interactions between the orbitofrontal cortex (OFC) and amygdala as critical for appropriate reward devaluation performance [3–6]. If similar lesions are restricted to the dorsolateral or ventrolateral prefrontal cortices, however, monkeys are not different from controls when tested on reward devaluation tasks [7, 8], indicating that the OFC, in particular, is the primary prefrontal cortical area underlying devaluation function. In agreement with these lesion studies, OFC area 11/13 volumes were shown to specifically correlate with reward devaluation performance, but not with performance on a reversal learning task [1] - a behavior that remains intact following lesions to the amygdala-OFC network [4, 8, 10]. Within the context of reward devaluation, the amygdala is thought to update reward-value information, while the OFC appears to use this information to represent and update outcome expectancies used to guide decision making behavior [4, 11].

Anatomical tract-tracing studies in nonhuman primates suggest that the OFC and amygdala are monosynaptically and bidirectionally connected via at least two separate white-matter tracts: the uncinate fasciculus (UF) and ventral amygdalofugal (AF) pathways [12–15]. The UF pathway uses a lateral route that courses through the anterior aspects of the temporal-frontal junction [16]. In addition to the fibers connecting the amygdala and OFC, the UF pathway also connects ventral prefrontal cortical regions with numerous other anterior temporal lobe structures, including the inferior temporal cortex and temporal pole. Furthermore, fibers within the UF pathway are known to connect various ventral prefrontal cortical regions with one another [14]. Compared to the UF pathway, amygdala-OFC fibers contained within the AF pathway project medially from the temporal lobe to join thalamic and brainstem fibers that project through the anterior segment of the internal, external, and extreme capsules to innervate much of the prefrontal cortex, including the OFC [14]. Due largely to methodological limitations that restrict the ability to isolate specific white-matter tracts for experimental manipulation, it remains unclear whether the OFC-amygdala interactions that occur via these two disparate projection systems are functionally distinct or redundant with respect to cognitive functions known to require interactions between these brain structures.

One method of studying the function of distinct whitematter pathways is to correlate behavioral or physiological measures of brain function with quantitative estimates of white matter condition using diffusion magnetic resonance imaging (dMRI) approaches [17–20]. For example, a common dMRI method, diffusion tensor imaging (DTI), can be used to measure the directional dependence of water diffusion in the brain [21–23]. Fractional anisotropy (FA) is a scalar measure obtained from DTI where 0 < FA < 1.0, and higher values indicate higher anisotropy [24]. Anisotropy can be particularly high along myelinated fiber tracts, and FA has been used clinically to assess the microstructural condition of white matter structures in both normal and pathological conditions [25, 26]. In humans and monkeys, FA estimates derived from multiple white-matter tracts, including the UF pathway have shown age-related reductions that relate to certain aspects of cognitive function [20, 27, 28]. For example, FA decreases in the superior longitudinal fasciculus and cingulum bundle of aged macaque monkeys correlate with age-related impairments in executive function [20].

How age-related changes in FA are associated with alterations in an older animal’s ability to update reward value remains unclear. Thus, the primary aims of the current study were to address the unresolved questions of 1) whether the FA associated with OFC-amygdala connectivity declines with age in monkeys as it appears to in humans, 2) whether the FA of the white-matter connecting these two structures correlates with behaviors that require intact amygdala-OFC interactions, particularly reward devaluation, and 3) whether FA values from anatomically distinct fiber bundles connecting the OFC and amygdala correlate with different aspects of reward devaluation. Here, we have used DTI as well as modelfree High Angular Resolution Diffusion Imaging (HARDI) methods [29] to generate FA and fiber orientation distribution (FOD) maps, respectively. Probabilistic tractography was carried out between the OFC and amygdala to quantitatively characterize the UF and AF projections separately in adult and aged macaques. In addition to imaging, all animals were behaviorally characterized on object-based and object-free versions of a reward devaluation task, as well as on a concurrent object reversal learning paradigm [1, 20, 31].

## Materials and Methods

### Subjects

Six adults (range: 11.25 – 15 years; mean: 13.3 years) and five aged (range: 24.25 – 30.8 years; mean: 26.0 years) female bonnet macaques *(Macaca radiata)* participated in this study, and were the same animals reported in [1]. Each animal received semiannual health assessments from the University of Arizona’s (Tucson, AZ) veterinary staff. No monkey presented with health concerns prior to or during the time of testing. The monkeys were all pair-housed in a humidity- and temperaturecontrolled vivarium with *ad libitum* access to food and water and a 12-hour light-dark cycle. All animals underwent behavioral shaping to tolerate transport in a specialized nonhuman primate holding box (50.8 cm x 31.1 cm x 40 cm) used to move them from the home vivarium to the behavioral testing apparatus (see below). The experiments described here all adhered to guidelines established by the National Institutes of Health and were approved by the Institutional Animal Care and Use Committee at the University of Arizona.

### Testing apparatus and stimuli

Behavioral testing was conducted in a modified Wisconsin General Testing Apparatus (WGTA; [32]). The WGTA is composed of a holding box where animals reside during testing. At one end of the box, vertical bars separate the monkeys from a tray that contains three equally spaced wells used for stimulus delivery and food reward administration. Experimenters could control the animals’ access to and visibility of stimulus objects via the use of a wooden guillotine door. A one-way mirror separated the monkeys from the experimenters, which allowed the animals’ performance to be monitored without detection. Plastic toy objects of comparable size (~8 cm^3^) were used as stimuli and rewards consisted of dry and fresh fruit, vegetables, and sugar free candy.

### Behavioral procedures

**Figure 1:**
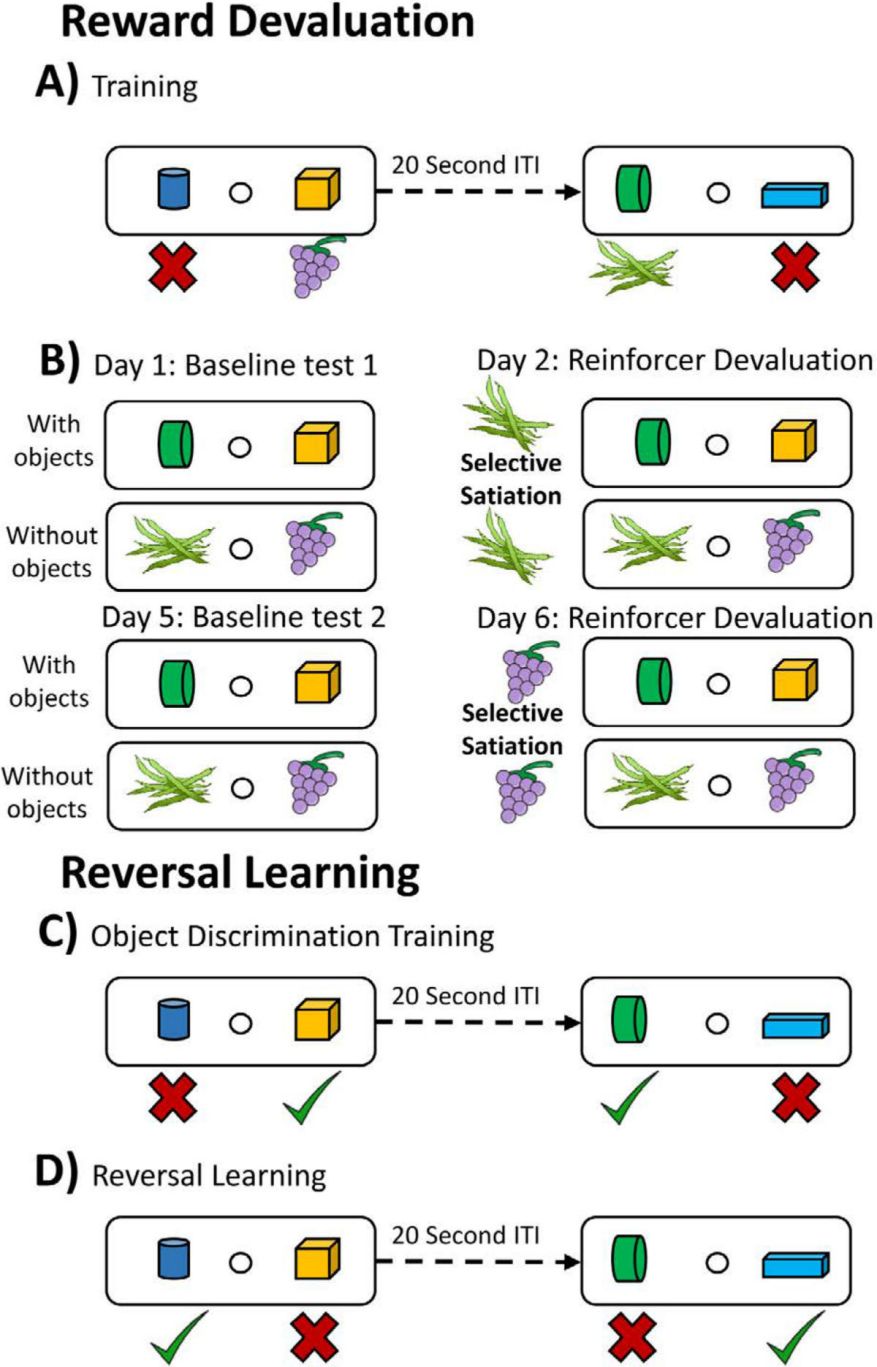
Schematic diagrams of the reward devaluation and reversal learning tasks. **A)** Prior to reward devaluation testing, all animals were trained on an object discrimination task with 40 different object pairs One object in the pair was always rewarded and the other was never reinforced. Half of the object pairs were rewarded with Food 1 (grapes in the schematic), while Food 2 (green beans in the schematic) was always used to reward the other half. The monkeys were all trained to a criterion performance level of 90% over 5 consecutive testing days, after which the animals participated in the reward devaluation test sessions (see below). **B)** During the reward devaluation testing with objects, only the rewarded objects from the object discrimination training sessions (i.e., associated with Food 1 and Food 2) were used. On object-free sessions, monkeys were allowed to directly select food reinforcement without using object-reinforcement associations. On Day 1, all animals underwent a baseline test with no satiation treatment (top-left panel). On Day 2, testing was preceded (~10 minutes) by a selective satiation of either Food 1 or Food 2 (top-right panel), which serves to de-value one of the rewards relative to baseline testing days. Baseline testing and satiation testing were repeated on Days 5 and 6, with the exception that the animals were satiated with the opposite food reward from Day 2. **C)** Reversal learning procedures - During object discrimination training, all monkeys learned 40 object pairs as in the first object discrimination task (A) to a criterion of 90% over 5 consecutive days. In this version of the object discrimination task there was no object-reinforcement pairing to a specific food. **D)** After criterion was reached, the rewarded and un-rewarded objects in the pairs learned in C were switched, requiring animals to alter their behavior in line with the new reinforcement contingencies. Testing continued until the animals reached 90% performance for 5 consecutive sessions after the reversals.

The reward devaluation and object reversal learning tasks used in this study have been described previously in detail [1, 30]. Schematic depictions of each task are found in figure 1.

#### Reward devaluation:

Briefly, all monkeys completed a 14-day food preference testing paradigm (described in [3]) where animals were presented all the possible food pairing combinations from the six following foods: dried cranberries, grapes, golden raisins, pears, carrots, and fruit snacks. Monkeys were allowed to select only one food in the pairing, and this selection was recorded as the preferred food in the pairing. The monkeys repeated this procedure for 30 trials a day over 14 days to establish each individual monkey’s food preference. The proportion of times that any individual monkey encountered each food combination was calculated, and the top two selected foods were used as Food 1 and Food 2 (for the selective satiation procedure described below). Therefore, a different Food 1 and Food 2 were used for every animal to account for individual food preferences.

Following food preference testing, the monkeys were trained on a set of 40 distinct object discrimination (OD) problems where animals were required to learn which item in an object pair is associated with a food reward. In this task two objects are placed over the outer wells of the WGTA, and the well under one item is baited with one of the six food rewards listed above, while the other well is not. The animals were allowed to displace only one object and retrieve the food reward if the item selected was the baited one. A 20 second inter-trial interval was used between object pairs. Animals completed 40 trials a day, therefore each object pair was encountered only once within a testing session. Half of the rewarded objects were rewarded with food 1, and the other half were rewarded with food 2, and the reward assignments were held constant across days, thus a secondary association between rewarded objects and food type existed. Monkeys were trained on this task to a performance criterion of 90% over 5 consecutive days (180/200 trials).

After reaching performance criterion, the monkeys completed the devaluation task, which tests the animal’s ability to use the predicted value of food reward to guide choices that maximize reward value. This task is composed of 4 test sessions: 2 baseline sessions, 1 session with devalued food 1, and 1 session with devalued food 2. In each of these sessions, only the rewarded objects from the OD training phase were used, and items were paired so that one was rewarded with food 1 and the other with food 2. The left-right position of the food 1- and food 2-rewarded objects was randomized using the ‘randperm’ function in MATLAB. The monkeys were required to select only one of the two objects and retrieve the food reward under it. Baseline sessions consisted of the monkeys performing the task without receiving any food in the morning prior to testing. The day following this baseline session the animals performed the same task ten minutes after undergoing a selective satiation procedure for food 1 or food 2 [3]. A second baseline testing session followed the selective satiation sessions.

After finishing the devaluation task with objects, the monkeys underwent an additional 4 days of reward devaluation testing without objects. In this test, the food rewards were presented without the objects, which serves as a control for the ability of satiation to modify food preferences since it eliminated the monkey’s need to use the secondary associations formed between the objects and the type of food reward. As in the reward devaluation with objects protocol, all monkeys underwent 2 baseline sessions and two satiation sessions (food 1 and food 2).

The effect of reward devaluation was quantified using a difference score, which was defined as the change in choices of each object/food type (object/food 1 and object/food 2) in selective satiation sessions relative to baseline sessions. The difference scores from the two satiation sessions were summed into a single value and are referred to simply as ‘difference score’ throughout this manuscript. Since animals performed 20 trials per session, and the difference score is the sum of two selective satiation procedures, the theoretical range that this index can take is [−40,40]. Negative values indicate a preference for selecting the devalued object during the selective satiation session relative to baseline, and positive values indicate a preference for the non-devalued object.

#### Reversal learning task:

After completing the reward devaluation tests, the monkeys were given a 2–3 week break from any form of cognitive testing. Following this break the animals were re-trained on the 40 object-pair OD task with a new library of objects. Again, a 20 second inter-trial interval was used. Once reaching the same 90% performance criterion over 5 consecutive days, the reward contingencies were reversed such that the previously unrewarded object became rewarded and vice versa. The monkeys continued to perform the task after reward-contingency reversal until they again performed at 90% over 5 consecutive days.

The effects of reward-contingency reversal were quantified using a state-space model of the binary trial-response data. This analysis has been described in detail elsewhere [30, 33]. Briefly, this modelling approach uses Bernoulli observation models that output a probability density function corresponding to the likelihood that an animal will select the correct answer on any given trial. The mode and 95% confidence interval of the probability function is then used to create a learning curve. The estimated learning trial was defined as the first trial that the lower bound of the 95% confidence interval exceeds and remains above chance (50%) for the remainder of the experiment. This value is referred to simply as the ‘learning trial’ throughout this manuscript.

### Image acquisition protocol

Prior to imaging, each monkey was anaesthetized with an intramuscular injection and was given an intravenous catheter for fluids and intubation as described in [1]. This ensured that there was no subject motion during the imaging. All monkeys were anesthetized with intramuscular injections containing midazolam (0.15 – 0.2 mg/kg), ketamine (1.5 – 2.0 mg/kg), and DexMedetomidine (0.007– 0.01 mg/kg), with older animals receiving the lower ends of these dose ranges. After sedation, the monkeys were intubated, and anesthesia was maintained using 2–3% sevofluorane delivered through an MRI-compatible vaporizer.

All MR images were acquired on a 3T GE (General Electric, Milwaukee, WI) Signa scanner, using a body coil for the radio frequency excitation and an eight-channel head coil for reception. The monkeys were imaged in a “sphinx” position, placed in a customized MRI-compatible stereotactic head holder with mouth, orbit and ear bars within the receiver coil. A vitamin-E capsule was placed near the right ear of the monkeys to be able to differentiate the right side of the image from the left. A T2-weighted reference scan of the whole brain was performed using a fast spin-echo sequence with the following parameters: TR = 3916 ms, TE = 74 ms, flip angle = 90°, section thickness = 1.4 mm, in-plane resolution = 0.7 mm × 0.7 mm, acquisition matrix = 256 × 256 (FOV = 179 mm × 179 mm) with 4 repetitions of 47 continuous coronal sections. Images were resectioned from the 3D volume into coronal, sagittal and axial slices. Diffusion-weighted (DW) images were acquired using a single shot echo planar imaging (SS-EPI) sequence with the following parameters: TR = 12500 ms, TE = 72 ms, flip angle = 90°, number of averages = 2. 47 continuous coronal sections were acquired, section thickness = 1.4 mm, in-plane resolution = 1.4 × 1.4 mm, and acquisition matrix = 128 × 128 (FOV = 179 mm × 179 mm). The data were acquired over 51 diffusion directions in a HARDI sampling scheme over a single shell with a b-value of 1000 s/mm^2^. Six volumes with no diffusion weighting (b = 0 s/mm^2^) were also acquired.

**Figure 2:**
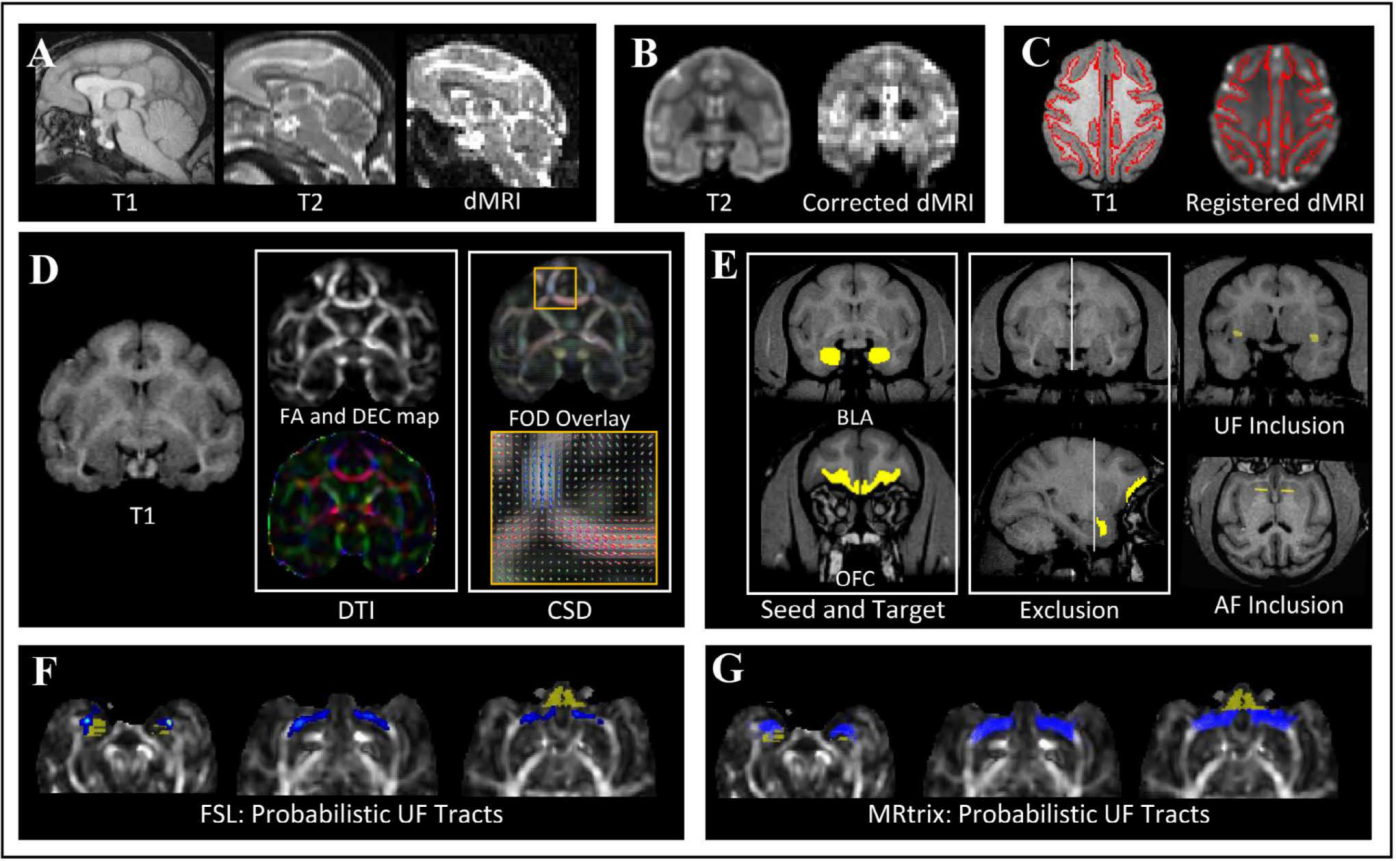
Image acquisition and processing pipeline. **A)** Representative sagittal section of a bonnet monkey brain imaged using a T1, T2 and Diffusion sequences.**B)** Coronal sections of a reference T2 image with a dMRI corrected for eddy current distortions and B0-field inhomogeneity using an iterative Gaussian Process-based registration and non-linear registration to reference T2 images, respectively. **C)** Axial sections of a reference T1 image and the corresponding dMRI image after registration. The gray matter-white matter interface is highlighted in red across the two images to show the effectiveness of the registration algorithm. **D)** Coronal parameter maps from DTI and CSD analysis. FA and a directional encoded color (DEC) map were obtained from DTI fitting. A FOD map, overlaid on FA, was obtained from CSD analysis. An expansion of the FOD map in the area near corpus collosum is shown in the inset. A T1 image of the same section is included for reference and colors in the image have their usual directions. **E)** ROI masks used in generating probabilistic fiber streamlines belonging to uncinate fasciculus (UF) and ventral amygdalofugal pathways (AF). **F, G)** Example UF streamlines generated by DTI (left) and CSD (right), overlaid on FA maps, shown for three axial sections moving from the amygdala towards orbitofrontal cortex (left to right in each insert). Yellow colored areas represent the amygdala and OFC seeds used, and blue areas represent the generated streamlines between regions.

High-resolution anatomical whole-brain T1-weighted images were acquired with a 3D inversion-recovery prepped spoiled gradient-echo sequence (IR-SPGR) with the following imaging parameters: TR = 8.1 ms, TE = 3.3 ms, TI = 500 ms, flip angle = 20°, acquisition matrix = 256 × 256 (FOV = 15.4 mm × 15.4 mm), in-plane resolution = 0.7 mm × 0.7 mm and section thickness = 0.7 mm with two repetitions of 86–100 coronal sections depending on the brain size. Representative T1, T2 and dMRI images are shown in figure 2A. The dMRI image corresponds to b = 0 s/mm^2^.

### Image pre-processing

DICOM images were converted to NIFTI format and T1, T2 and dMRI images were skull stripped using manually drawn masks in MRIcro (https://www.nitrc.org/projects/mricron). Eddy current distortions in dMRI images were corrected using an iterative Gaussian Process based registration in FSL [34, 35]. Distortions due to B0-field inhomogeneity were corrected using the TORTOISE software [36] by non-linearly registering the dMRI images to reference T2 images. Figure 2B shows a coronal section of a reference T2 and the corresponding dMRI image corrected for these distortions. Coil inhomogeneity was corrected using N4ITK bias correction software [37, 38] and noise in dMRI images was removed using a local principal component based noise removal algorithm described in [39].

The dMRI images were registered to T1 images using FSL’s Automated Segmentation Toolbox (FAST), followed by FSL’s Linear Image Registration Tool (FLIRT) and a Boundary Based Registration algorithm [40]. An axial section of a T1 image and the corresponding registered dMRI image is shown in figure 2C.

### Region of interest

Region of interest (ROI) masks for the amygdala and the orbitofrontal cortex were drawn manually by two observers on the high resolution T1 images as described in [1]. The ROI mask for amygdala consisted of the basolateral and basomedial nuclei. OFC volumes between the two raters were significantly correlated (r = 0.75, p < 0.0001), and were 10.6% different, and amygdala volumes were also significantly correlated (r = 0.80, p < 0.0001), and were 10.0% different; thus, the average volumes of each region were used. As several fiber tracts exist between the OFC and the amygdala, it was essential to restrict streamlines to specific white matter pathways to exclude other potentially confounding white matter pathways from being considered. As such, two exclusion ROI masks were drawn, one on a sagittal section separating the two hemispheres with the intent to exclude commissure fibers and the other on a coronal section posterior to the amygdala to exclude inferior longitudinal fasciculus fibers from interfering with the extraction of amygdala-OFC fibers. For UF extraction, an inclusion ROI mask was drawn on a coronal section in the region where the UF bundle is known to transition from the temporal to the frontal lobe as described in [12]. Representative masks for the UF regions of interest are shown on T1 images for a monkey in figure 2E. For AF extraction, an inclusion ROI mask was drawn on a coronal section to encompass the anterior aspect of the internal capsule [14], as also shown in figure 2E.

### Image processing and analysis

#### Diffusion tensor imaging analysis:

Fractional Anisotropy (FA) maps were generated from diffusion tensor fitting of the dMRI images in each subject’s native space. Figure 2D shows a coronal section of a T1 image and its corresponding FA and direction encoded color map. The colors represent the direction of the underlying fibers where red is left/right, blue is posterior/anterior and green is inferior/superior.

#### Constrained spherical deconvolution analysis:

A mean diffusion response function of a single fiber bundle was iteratively calculated from all monkeys as described in [41, 42]. The mean response function was de-convolved with the dMRI signal in each voxel to yield the underlying Fiber Orientation Distribution (FOD), a probability distribution of partial volumes of fibers in a voxel [43]. Figure 2D shows FOD overlaid on a coronal section of an FA map. The image in the box shows a zoomed-in region near the corpus callosum depicting voxels that contain a single fiber population as well as voxels that contain crossing fibers. Once again, the colors are indicative of the underlying fiber orientation.

### Tractography

For each subject, probabilistic streamlines were generated in the native space from the amygdala ROI mask towards the OFC using the exclusion and the inclusion ROI masks described above. The UF and AF streamlines were tracked separately in the two hemispheres. Two separate algorithms were used to generate streamlines. First, multi-tensor tractography was used to yield probabilistic white matter pathways of interest that can be used in tract-based analysis. Second, an algorithm utilizing FODs was used to quantify the number of streamlines in the fiber bundles of interest.

#### DTI-based tractography:

FSL’s Diffusion Toolbox, ProbtrackX, [44, 45] based on the Bayesian-estimation of diffusion parameters (BedpostX), was used to initiate 5000 streamlines from the amygdala ROI mask. Streamlines were propagated towards the OFC in the native space with a step size of 0.5 mm and a curvature threshold of 0.2. This yielded a connectivity distribution map of the UF or AF, where individual voxel intensities represent the number of tracts that pass through the voxel and successfully reach the OFC. The algorithm also outputs the ‘way-total’, which is the total number of tracks from the amygdala ROI mask that are successful in reaching the OFC via the inclusion ROI mask. The connectivity distribution maps were divided by the waytotal for normalization and voxels with intensity less than 40% of the 95^th^ percentile in the final weighted UF map were set to zero, similar to previously reported methodology [46]. Each voxel in this weighted mask is representative of the probability that the voxel belongs to UF/AF. Thus, while it is not accurate to say that the voxels extracted here only contain white matter connecting the OFC and amygdala, the probabilistic weighting of FA in this analysis gives a closer approximation of the FA associated with these two structures than an ROIbased approach is able to provide. Figure 2F shows a weighted UF mask in blue on three different axial sections moving from the amygdala towards the OFC, which is depicted in yellow.

For each subject, the weighted UF and AF masks was used to extract the FA along each pathway, and a mean value was computed per hemisphere to yield an FA index.

#### CSD-based tractography:

Tractography on the UF was also performed in MRtrix [47] using a 2^nd^ order integration over the fiber orientation distributions (iFOD2) algorithm [48]. From every voxel in the amygdala ROI mask, 2000 streamlines were initiated with a step size equal to half the voxel size and a curvature of 45 degrees. The streamlines propagate along the peaks of the calculated FODs in each voxel. Figure 2G shows probabilistic UF streamlines overlaid on three axial sections. The width of the streamlines is representative of the number of fibers.

The number of streamlines generated in each subject was divided by the corresponding amygdala volume and multiplied by average amygdala volumes across all subjects for normalization. This normalized quantity was used for comparisons between subjects.

### Statistical analysis

#### Behavioral analysis:

Behavioral data from the object reversal learning and reward devaluation tasks were analyzed using a 2-way ANOVA with age group (adult and aged) and epoch (OD and RL for the object reversal learning task; with object and without objects for the reward devaluation task) as factors. The alpha level for this analysis was 0.05. Mean and standard error of the mean values are presented before ANOVA statistics. Post-hoc age group comparisons were done using an unpaired t-test. A significance criterion of p < 0.05 was used, and p-values underwent Bonferroni correction for multiple comparisons.

#### Fractional anisotropy analysis:

FA estimates and numbers of generated tracks were analyzed using a 2-way ANOVA with age group (adult and aged) and hemisphere (right and left) as factors. Again, an alpha level of 0.05 was used. Post-hoc age group comparisons of for each hemisphere were done with an unpaired t-test. A significance criterion of p < 0.05 was used, and p-values underwent Bonferroni correction for multiple comparisons.

#### Relationships between imaging and behavioral data:

The relationship between imaging measures and cognition were statistically analyzed using a robust regression analysis. Briefly, this analysis is an alternative to least-squares regression analyses and is commonly used with small sample sizes and when outliers contaminate the data [49]. Intuitively, this method weights each data point by the inverse of the extent to which it is an outlier. In the imaging measure versus behavioral performance analysis, the imaging metrics were the independent variables and the behavioral performance measures were the dependent variables. In all cases a significance criterion of p < 0.05 was used.

## Results

### Behavioral results

**Figure 3:**
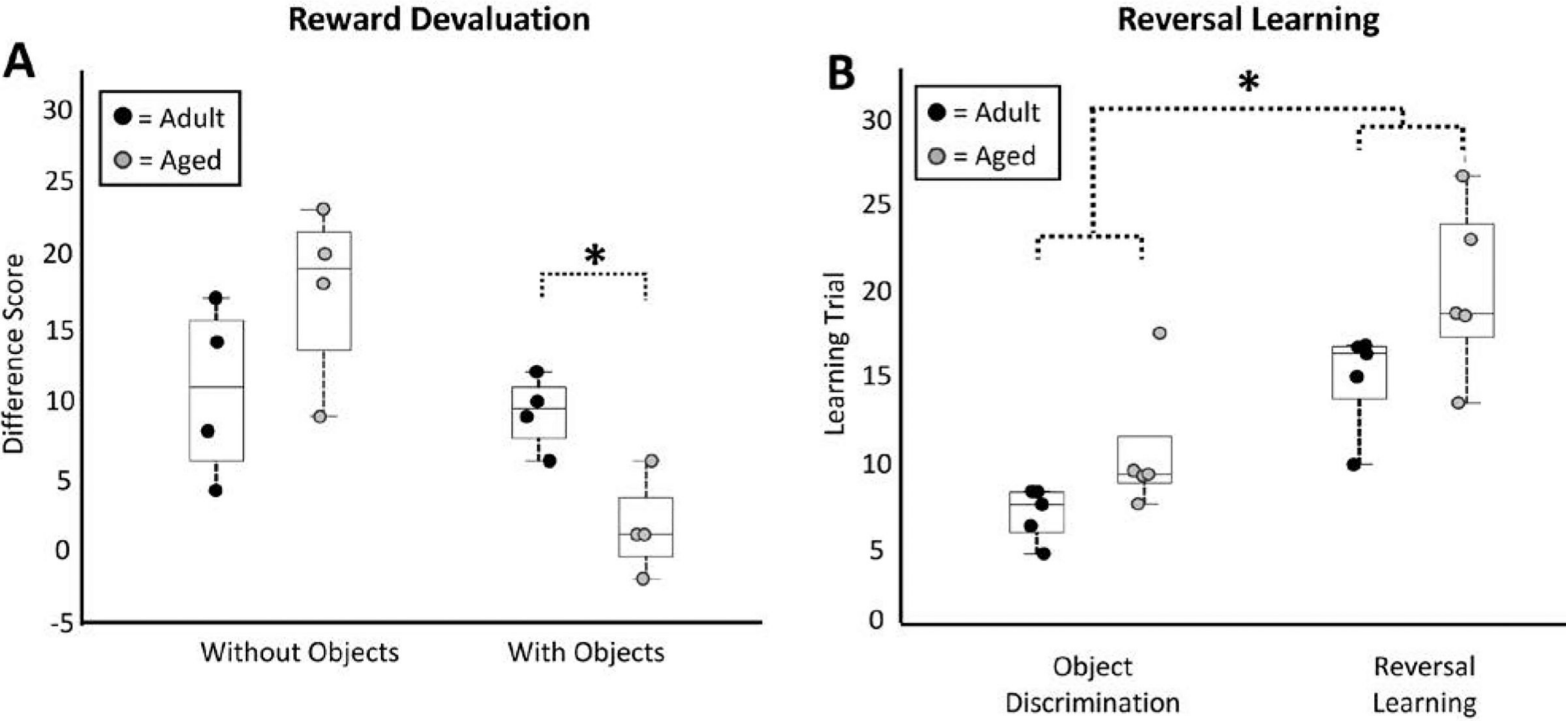
Reward Devaluation and Reversal Learning performance. **A)** Boxplots of difference scores of adult and aged animals on the reward devaluation task with and without object-associations. Boxes represent the middle 50% of the data, and horizontal lines mark the median of each distribution. Each filled circle indicates an individual monkey, with black circles representing adult animals and grey representing aged animals. Aged animals had lower difference scores on devaluation tests requiring the use of an object-reward association, but performance was not statistically different between groups on control sessions without objects. **B)** Boxplots of the number of trials that adult and aged monkeys required to learn the object discrimination and reversal learning tasks. Box-and-whisker plots are as in A. Aged monkeys required more trials to learn both the object discrimination and reversal learning components of the task. * = p < 0.05.

During the reward devaluation task, difference scores were higher during conditions without objects compared to conditions with objects (object-free: 14.13 +/− 1.98, objectbased: 5.38 +/− 1.49; 2-way ANOVA, F (1, 13) = 8.40, p = 0.01; Figure 3A). Difference scores did not differ between adult and aged monkeys in conditions without objects (adult: 10.75 +/− 2.39, aged: 17.50 +/− 2.70; t-test, p = 0.18, t = 1.61; Figure 3A). Aged animals did, however, have lower difference scores compared to adult monkeys in conditions that required animals to use object-reward associations to guide behavior (adult: 9.25 +/− 1.02, aged: 1.50 +/− 1.48; t-test, p = 0.04, t = −3.73; Figure 3A).

During the reversal learning task, both adult and aged monkeys required more trials to learn the reversal component of the task compared to the object discrimination phase (object discrimination: 8.88 +/− 3.38, reversal: 17.52 +/− 4.69; 2-way ANOVA, F (1, 17) = 30.74 p < 0.001; Figure 3B). On average, aged animals required more trials to learn the task (object discrimination adult: 7.09 +/− 0.63, object discrimination aged: 10.67 +/− 1.75, reversal adult: 14.97 +/− 1.19, reversal aged: 20.07 +/− 2.23; 2-way ANOVA, F (1, 17) = 7.76, p = 0.01; Figure 3B). Direct, post-hoc age comparisons of the OD and RL phases of the task revealed similar trends, although neither of these comparisons reached statistical significance (OD: t-test, p = 0.12, t = 1.91; RL: p = 0.11, t = 1.98).

### Diffusion-tensor imaging results

**Figure 4:**
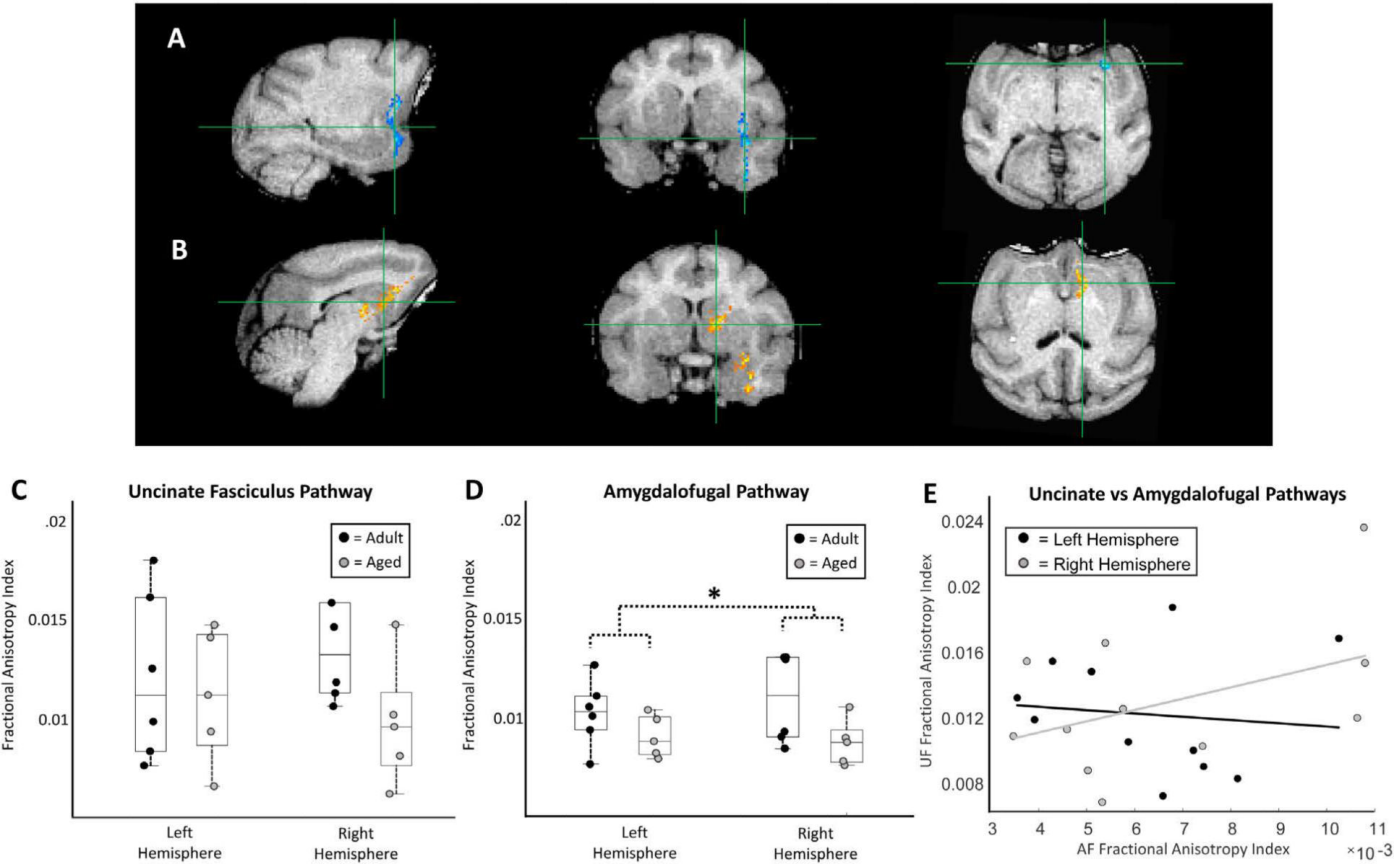
Uncinate fasciculus and ventral amygdalofugal pathway fractional anisotropy estimate. **A)** Representative probability map of the right hemisphere uncinate fasciculus, derived from probabalistic tractography in FSL, overlaid upon a T1-weighted MRI. **B)** Representative probability map of the right hemisphere ventral amygdalofugal pathway overlaid upon a T1-weighted MRI from the same animal depicted in A. **C)** Boxplots of uncinate fasciculus fractional anisotropy indices for each individual monkey separated by left and right hemisphere. Boxes represent the middle 50% of the data, and horizontal lines mark the median of each distribution. Each filled circle indicates an individual monkey, with black circles representing adult animals and grey representing the aged. Although not statistically significant, a trend towards reduced right hemisphere FA indices was evident in aged animals. **D)** Boxplots of amygdalofugal pathway fractional anisotropy indices for each individual monkey separated by left and right hemisphere. Boxplots as in C. Aged animals had significantly lower FA index scores than did adult. **E)** Scatter plot of fractional anisotropy indices from the UF and AF plotted against one another separately for each hemisphere. Grey dots represent data from the right hemisphere and black dots represent data from the left. There were no significant relationships found. * = p < 0.05.

Estimates of UF fractional anisotropy were not statistically different between adult and aged monkeys, nor was there a statistically significant hemispheric difference (left hemisphere adult: 0.013 +/− 0.0017, left hemisphere aged 0.012 +/− 0.0015, right hemisphere adult: 0.015 +/− 0.0019, right hemisphere aged: 0.010 +/− 0.0014; 2-way ANOVA, Age: F (1, 19) = 2.73, p = 0.12; Hemisphere: F (1, 19) = 0.16, p = 0.69; Figure 4C); although, on average, aged animals had reduced FA in the right hemisphere compared to adult (t-test: p = 0.11, t = −1.96; Figure 4C). Similarly, the number of estimated UF tracks generated via HARDI based fiber tractography did not differ between aged groups in either the left or right hemisphere (left hemisphere adult: 1.2240e+03 +/− 803.05, left hemisphere aged: 1.6574e+03 +/− 769.10, right hemisphere adult: 1.6327e+03 +/− 325.32, right hemisphere aged: 906.14 +/− 237.42; 2-way ANOVA -Age: F (1,19) = 0.06, p = 0.86; Hemisphere: F (1,19) = 0.04, p = 0.88). Estimates of AF fractional anisotropy, were significantly lower in aged animals compared to adult, although there also were no hemispheric differences (left hemisphere adult: 0.0070 +/− 6.7807e-04, left hemisphere aged: 0.0054 +/− 6.4284e-04, right hemisphere adult: 0.008 +/− 0.0012, right hemisphere aged: 0.0050 +/− 7.0198e-04; 2-way ANOVA, Age: F (1, 19) = 5.9, p = 0.0253; Hemisphere: F (1, 19) = 0.130, p = 0.719; Figure 4D).

In both hemispheres, UF and AF fractional anisotropy measures were not correlated with one another (robust regression: FA left hemisphere - p = 0.205, r = −0.05, t = −0.289; FA right hemisphere - p = 0.16, r = 0.474, t = 1.366; Figure 4E).

### Correlations of fractional anisotropy indices with reward devaluation and reversal learning

**Figure 5:**
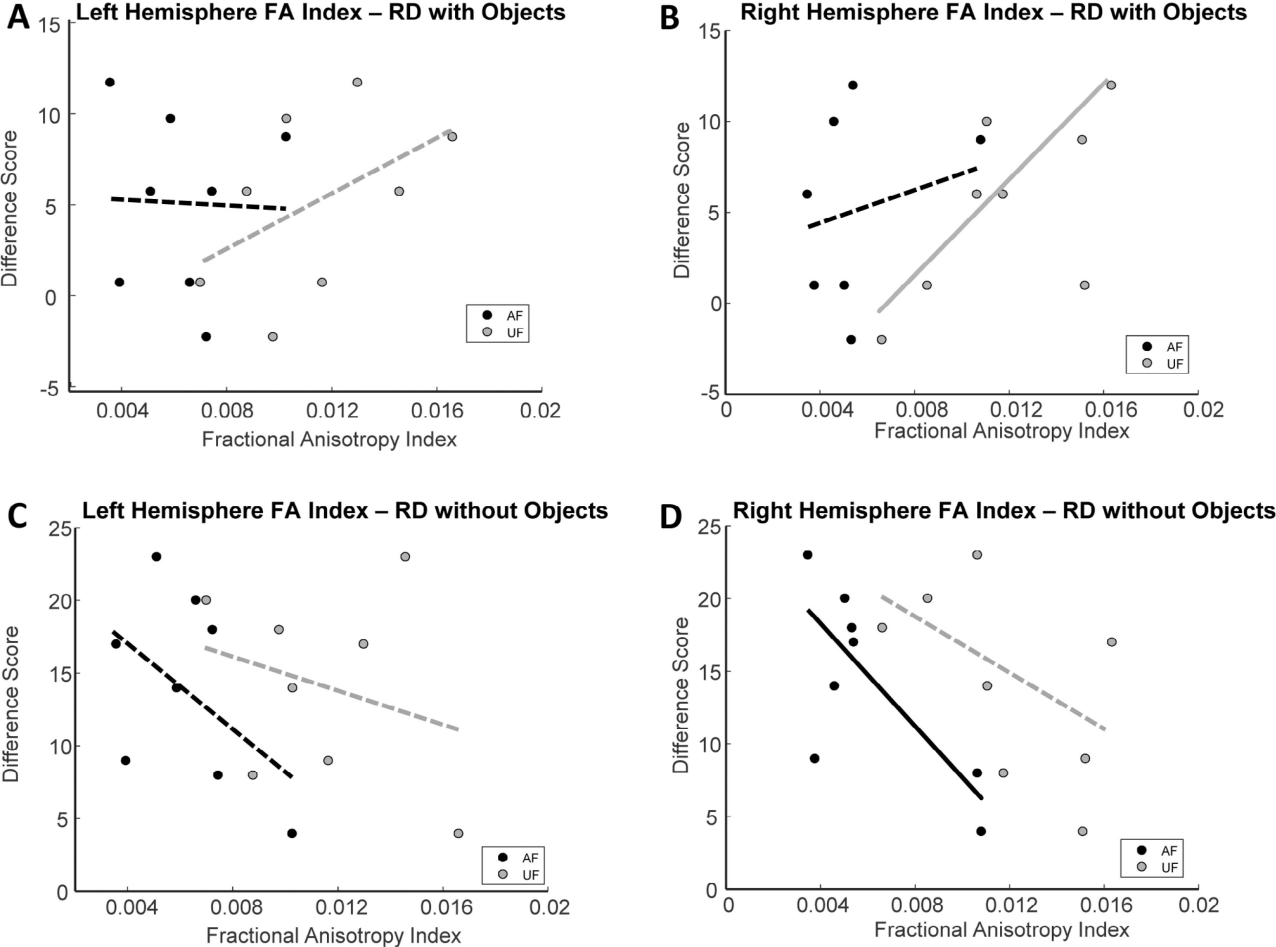
Relationship between uncinate fasciculus and amygdalofugal pathway fractional anisotropy (FA) indices with reward devaluation performance. In all plots, black dots represent data extracted from the amygdalofugal pathway and grey dots represent data from the uncinate fasciculus. Dotted trend lines indicate a statistically non-significant correlation, and solid trend lines indicate statistically significant relationships. Scatter plots of **A)** left hemisphere and **B)** right hemisphere FA index values from each pathway plotted against object-based reward devaluation performance scores. Right hemisphere uncinate fasciculus FA indices were significantly associated with the object-based version of the task. Scatter plots of **C)** left hemisphere and **D)** right hemisphere FA index values from each pathway plotted against object-free reward devaluation performance scores. In this version of the task, only the right hemisphere amygdalofugal pathway FA scores were significantly negatively associated with performance.

Fractional anisotropy from the UF and AF of each hemisphere were related to performance on both object-based and object-free versions of the reward devaluation task. In the left hemisphere, the fractional anisotropy indices derived from both the UF and AF did not significantly correlate with the object-based version of the reward devaluation task (robust regression - UF: p = 0.243, r = 0.50, t = 1.296; AF: p = 0.9398, r = −0.047; t = −0.0787; Figure 5A). Right hemisphere UF fractional anisotropy indices were significantly correlated with performance on the object-based version of the task (robust regression - p = 0.013, r = 0.634, t = 3.462; Figure 5B), whereas these same measures extracted from the right hemisphere AF did not (robust regression - p = 0.564, r = 0.269, t = 0.610; Figure 5B).

Left hemisphere fractional anisotropy indices for the UF and AF were also not significantly associated with the objectfree version of the reward devaluation task (robust regression - UF: p = 0.559, r = −0.255, t = −0.319; AF: p = 0.25, r = −0.482, t = −1.265; Figure 5C). In the right hemisphere, however, fractional anisotropy indices derived from the AF pathway showed significant negative correlations with performance on the object-free version of the reward devaluation task (robust regression - p = 0.035, r = −0.728, t = −2.709; Figure 5D), while the same estimates from the UF were not significantly correlated (robust regression - p = 0.17, r = −0.550, t = −1.532; Figure 5D).

**Figure 6:**
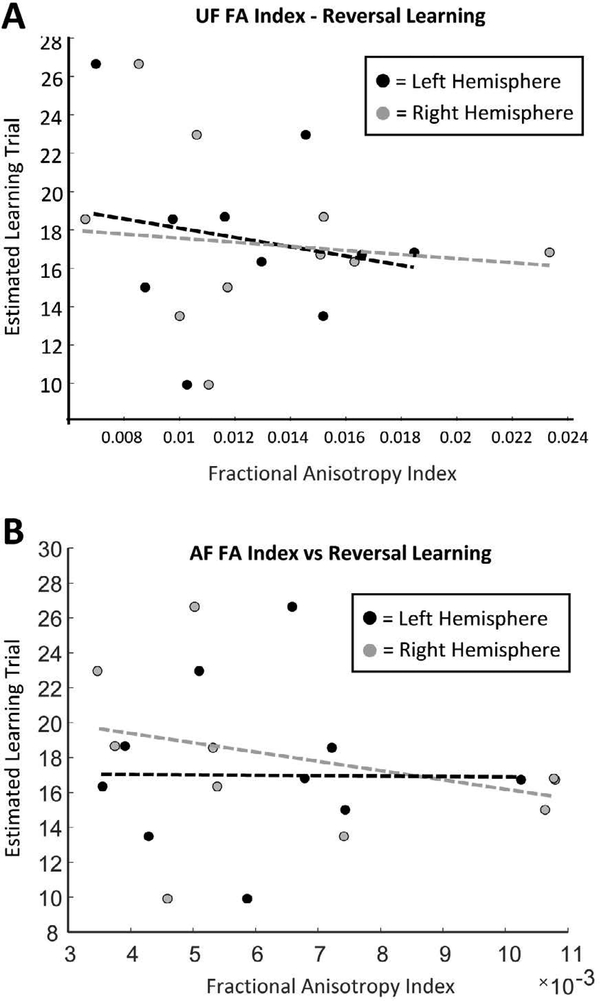
Relationship between uncinate fasciculus and amygdalofugal pathway fractional anisotropy (FA) indices with reversal learning performance. In both plots black dots indicate data extracted from the left hemisphere and grey dots represent data from the right. **A)** Uncinate fasciculus FA indices were not significantly associated with the estimated learning trial on the reversal learning task. **B)** Similarly, amygdalofugal pathway FA values were not significantly associated with the estimated learning trial on the reversal learning task.

Left and right hemisphere UF fractional anisotropy estimates did not correlate with the estimated learning trial of the reversal learning task during either the object discrimination learning phase (robust regression; left hemisphere: p = 0.302, r = −0.287, t = 1.103; right hemisphere: p = 0.786, r = −0.217, t = 0.281) or the reversal phase (robust regression; left hemisphere: p = 0.631, r = −0.228, t = −.499; right hemisphere: p = 0.762, r = −0.200, t = −0.314; Figure 6A). Similarly, left and right hemisphere AF fractional anisotropy indices did not correlated with either the object discrimination or reversal component of the reversal learning task (robust regression; OD - left hemisphere: p = 0.447, r = −0.103, t = −0.800; right hemisphere: p = 0.529, r = −0.263, t = −0.687; RL - left hemisphere: p = 0.992, r = 0.029, t = 0.0098; right hemisphere: p = 0.364, r = −0.311, t = −0.963; Figure 6B).

## Discussion

The first major aim of the present study was to determine whether the fractional anisotropy (FA) of two prominent white matter tracts connecting the amygdala and orbitofrontal cortex (OFC) differs across age in macaque monkeys. Comparisons of FA indices extracted from the ventral amygdalofugal (AF) pathway were in fact lower in aged monkeys compared to adults, and a similar trend was noted in the right hemisphere UF pathway (Figure 4). Second, the present study set out to determine whether FA indices from the AF and/or UF pathways relate to a monkey’s ability to perform cognitive operations that require interactions between the amygdala and OFC. Indeed, FA values from both white matter tracts significantly correlated with performance on a reward devaluation task known to require intact amygdala-OFC interactions (Figure 5), whereas FA values from neither anatomical pathway correlated with performance scores on a concurrent reversal learning task for which there is evidence does not require these structures [3–6, 9] (Figure 6). Together these results indicate that age-related alterations in the state of the white matter tracts associated with amygdala-OFC connectivity has functional consequences specifically on cognitive functions that emerge due to network interactions between these brain structures.

### Age-related fractional anisotropy decreases in white matter associated with amygdala-OFC connectivity

In humans, studies that have used DTI methods to investigate age-associated changes in FA have generally reported linear decreases in healthy adults starting around the age of 20 [19, 50, 51]. Importantly, these reductions in the FA are not uniform across the brain, but rather vary significantly between distinct white matter tracts [50]. One regional pattern that has consistently emerged is an anterior-posterior gradient of age-related FA decline, with white matter in more anterior regions showing larger decreases regardless of the fiber tract’s medial-lateral position [50, 52–56]. Since prefrontal cortical neuronal networks are known to be critical for various aspects of cognition [57–60] and are vulnerable to age-associated dysfunction [61, 62], these observations suggest that changes in the condition of forebrain white matter tracts play a significant role in the emergence of age-related cognitive decline.

Nonhuman primate models of cognitive aging have been used for decades because of the advantages that these animals provide over other model species [for review see 63]. One such benefit is that monkeys and humans share numerous homologous brain regions and patterns of white matter connectivity between structures, making interspecies comparisons relatively straightforward compared to other animal models [64–66]. Like in humans, the white matter associated with frontal cortical regions in monkeys has shown a similar vulnerability to age-associated reductions in FA. For example, Makris et al. showed significantly reduced FA in cortico-cortical fiber pathways associated with the frontal lobes (specifically within the superior longitudinal fasciculus II, cingulum bundle, and anterior segment of the corpus collosum), but not in the more posterior corticospinal tracts [20]. In the present study, the observed significant decline in FA indices along the AF pathway (Figure 4), and a trend for a decline in the UF pathway, further supports observations that white matter tracts innervating the frontal cortex are particularly impacted by aging. More support for the idea that white matter in frontal brain regions is vulnerable during aging comes from unpublished data acquired from the thalamic radiations of these same monkeys. Specifically, tracts directed towards the prefrontal cortex have significantly lower FA indices in older animals, while the more posterior thalamic radiations innervating auditory regions in the temporal lobe are relatively preserved in older monkeys. Together, these findings suggest that the regional distribution of white matter change in aged monkeys is reminiscent of the patterns observed in older humans [50, 55, 56].

### FA indices from the uncinate fasciculus and amygdalofugal pathways correlate with reward devaluation behavior

Relationships between FA and brain function have been regularly observed using DTI methods in human subjects [50, 67–69]. For example, Schulte et al. assessed the relationship between interhemispheric transfer speed of visuomotor information and FA measurements from various commissural white matter sources [67]. Greater transfer times, which are indicative of poorer performance on this task, were associated with lower FA values in the genu of the corpus collosum. This suggests that inter-individual differences in commisural white matter microstructure influence interhemispheric processing. Interestingly, a multiple regression model using FA values from the genu, body, and splenium portions of the corpus collosum identified only the genual region as a significant predictor of performance on the interhemispheric transfer task [67]. These results suggest that differences in white matter microstructure have functional consequences specifically on brain operations that require the structures associated with the fiber tract under investigation. In further support of this hypothesis, FA reductions in cortico-cortical white matter pathways innervating the prefrontal cortex of aged monkeys were significantly associated with tests of executive function, but not with object recognition abilities thought to rely heavily on medial temporal lobe function [20].

A central aim of the current study was to test the hypothesis that the FA of the white matter associated with amygdala-OFC connectivity relates specifically to behaviors known to require these brain regions, and not to tasks that do not. It was therefore imperative to select behaviors that both do and do not probe the integrity of amygdala-OFC neuronal networks.As mentioned previously, reward devaluation performance in monkeys is significantly impaired following lesions that disrupt amygdala-OFC interactions, whereas performance on the concurrent version of a reversal learning paradigm remains intact following similar experimental manipulations [3, 5, 6, 9, 31]. Despite the distinct neural underpinnings of these two behaviors, aged macaques are impaired on both tasks relative to younger animals [1, 30, 63, 70, 71] (Figure 3A). The present observation that FA indices from the UF and AF relate only to the reward devaluation paradigm, but not to the reversal learning paradigm, supports the conceptualization that regional declines in white matter condition may underlie specific, rather than more global aspects of cognitive decline.

### Uncinate fasciculus and amygdalofugal pathway fractional anisotropy indices are differentially related to reward devaluation behaviors

Traditional tests of reward devaluation [2, 3] examine an animal’s ability to use previously established objectreinforcement associations. In this case selective satiation procedures are used to manipulate an animal’s desire to obtain a specific type of reward. In addition to this **object-based** version of the reward devaluation paradigm, the monkeys in this study also underwent an **object-free** version of the task where the reinforcement choices were directly presented to the animal (see Methods; Figure 1). These object-free tests of reward devaluation were administered to control for the possibility that the satiation procedures used may differentially modify food preferences in adult versus older animals [1]. During the object-based version of the task, the aged monkeys examined here selected the devalued food option more often than did the adult animals (Figure 3A), suggesting that aging is associated with a diminished ability to use objectreinforcement associations to guide reward-seeking behaviors. In contrast, adult and aged monkeys performed similarly on the object-free version of the task (Figure 3A), suggesting that the deficits noted in the object-based version are not due to differential effects of satiation between age groups.

A dissociation emerged in the relationships between performance on object-based and object-free versions of the reward devaluation task and FA indices extracted from the UF and AF pathways. In the case of the object-based version of the task, FA measures from the right UF pathway were significantly positively related to the tendency for monkeys to select the object associated with the more valuable reinforcement option (Figure 5B). Note that while the relationship between left hemisphere UF pathway anisotropy values and difference scores on the object-based version of the task did not reach statistical significance, there was a positive correlation between the two (r = 0.50). In contrast, AF pathway values from both the left and right hemispheres were not related to object-based reward devaluation performance. For the object-free version of the task, right hemisphere FA indices from the AF pathway were significantly negatively associated (Figure 5D). Note that although not statistically significant, FA indices from the left hemisphere AF pathway and the right hemisphere UF pathway also showed prominent negative correlations with the objectfree version of the task (r = - 0.48; r = - 0.55, respectively).

The positive association between object-based reward devaluation performance and FA indices from the right hemisphere UF pathway suggests that amygdala-OFC interactions via this pathway may play a greater role than AF pathway connections in reward seeking behavior dependent on the use of previously established stimulus-reward associations. This proposed functional dissociation between the UF and AF pathways with respect to reward devaluation behavior is well supported in the context of the known functional specializations of the medial and lateral OFC. In macaques, ablations restricted to lateral OFC areas 11/13 and 12 cause deficits in a monkey’s ability to attribute reward credit to appropriate stimuli (reward credit assignment) and in the rapid updating of object values [72–74]. In contrast, monkeys with lesions restricted to the medial OFC show no such deficits, but rather are impaired in reward-guided decision making as reflected by a tendency to perseverate on previously rewarded stimuli during extinction tasks [72, 73]. Since amygdala-OFC fibers contained within the UF pathway are known to preferentially terminate in the lateral OFC, whereas the fibers contained within the AF pathway preferentially terminate in the medial OFC [14, 75], the present findings that objectbased reward devaluation performance relates only to FA indices extracted from the more lateral UF pathway fits into this framework of functional specialization across the mediallateral axis of the OFC.

The negative association between FA indices extracted from the AF pathway and performance on the object-free version of the task might be explained by the behavior of the older animals. That is, although statistically not significant, aged animals on average selected the more valuable food option in the object-free version of the task more often than did adult animals (Figure 3A). A more detailed analysis of performance across individual sessions revealed that aged animals selected the higher-value reinforcement option more than did adult animals, but only during the second half of the testing sessions (t-test; 1^st^ half - p = 0.178, t = 1.9060, df = 9; 2^nd^ half - p = 0.015, t = 3.43, df = 9). This difference appeared to emerge due to a greater tendency for the adult monkeys to select the devalued food reinforcement later in the session (2^nd^ - 1^st^ half differences scores: adult: −1.33, aged: 1.6). These findings may indicate that adult animals modified their representation of the reward value with experience from repeated exposures, whereas older animals may use the reward value established at the beginning of testing session throughout. If this interpretation is correct, then lower FA values in the AF pathway were related with poorer performance on the object-free reward devaluation task. Since the OFC is thought to receive reward-value information from the amygdala to update behavioral strategies [4, 11], these findings may reflect a tendency for animals with more poorly organized amygdala-OFC connectivity to behaviorally perseverate on certain reward values. Furthermore, these findings could indicate that amygdala-OFC network interactions that occur via the AF pathway play prominent roles in updating reward values used by the frontal lobe to drive reward seeking behaviors.

### Diffusion tensor imaging (DTI) and constrained spherical deconvolution (CSD) tractography

Another aim of this study was to directly compare two separate approaches for estimating the white-matter integrity - traditional DTI imaging and model-free CSD probabilistic tractography (see Methods; Figure 2). Traditional DTI approaches suffer from several drawbacks, an important one being the inability to model crossing fibers within a voxel [76], which can lead to underestimations in anisotropy. Alternative techniques, such as HARDI and CSD, that acquire images with diffusion weighting along many more directions than traditional DTI, allow a more accurate estimation of diffusion, theoretically yielding more precise estimates of white matter microstructure. In this study both estimation techniques yielded similar, statistically significant positive relationships between right hemisphere FA indices and object-based reward devaluation performance.

## Conclusion

The present study reports age-associated reductions in the fractional anisotropy of white matter associated with amygdalaorbitofrontal cortex connectivity in macaque monkeys. Fractional anisotropy indices from the right hemisphere uncinate fasciculus pathway were significantly associated with poorer performance on an object-based reward devaluation task. On the other hand, fractional anisotropy measures from the more medial amygdalofugal pathway of the right hemisphere were significantly associated with poorer performance on an object-free version of this task. Together these data suggest that the fractional anisotropy of the white matter associated with amygdala-orbitofrontal cortex connectivity declines with age in macaque monkeys, and age-related declines in different anatomical tracts connecting these structures are related to specific aspects of reward-guided behavior.
